# Immune cell dynamics and the impact on the efficiency of transvascular antitumor interventional therapies in hepatocellular carcinoma patients

**DOI:** 10.3389/fimmu.2024.1450525

**Published:** 2024-10-08

**Authors:** Yuan-Dong Sun, Hao Zhang, Yuan-Min Li, Chun-Xiao Zhou, Jian-Jun Han

**Affiliations:** ^1^ Department of Interventional Radiology, Shandong Cancer Hospital and Institute Affiliated Shandong First Medical University and Shandong Academy of Medical Sciences, Ji’nan, China; ^2^ Key Laboratory of Transplant Engineering and Immunology, National Health Commission (NHC), Frontiers Science Center for Disease-Related Molecular Network, West China Hospital, Sichuan University, Chengdu, China; ^3^ Division of Gynecologic Oncology, University of North Carolina at Chapel Hill, Chapel Hill, NC, United States

**Keywords:** hepatocellular carcinoma, white blood cells, lymphocyte subpopulations, progressive free survival, disease control

## Abstract

**Objective:**

This study investigates the impact of transvascular antitumor interventional therapies on immune cell dynamics and its correlation with disease control and progression-free survival (PFS) in hepatocellular carcinoma (HCC) patients.

**Methods:**

A single-center observational case-control study was conducted with 119 HCC patients. Transvascular antitumor interventional therapy were administered based on patient-specific evaluations. Peripheral blood samples were collected before and within 28 days after the first treatment to analyze lymphocyte subsets and other immune cells.

**Results:**

Higher counts of total white blood cells (WBCs), lymphocytes, monocytes, and basophils were significantly associated with disease control rate. Subgroup analysis revealed that abnormal BMI, diabetes, infection, and multiple lesions were significantly associated with T cell abnormalities. Age, abnormal BMI, hypertension, and abnormal AFP were linked to total T cell abnormalities. NK cells, B cells, Th cells, Tc/Ts cells, and CD4/CD8 ratios did not show significant differences in PFS probabilities.

**Conclusion:**

Higher counts of WBCs, lymphocytes, monocytes, and basophils, play a crucial role in the effectiveness of HCC interventional therapy.

## Highlights

- Higher WBC, lymphocyte, monocyte, and basophil counts correlate with successful HCC treatment outcomes.
- T cell abnormalities associate with clinical factors like BMI, diabetes, infection, and multiple lesions.
- Total T cell abnormalities link with age, BMI, hypertension, and AFP levels in HCC patients.
- Certain immune cell abnormalities do not significantly influence progression-free survival in HCC.
- Personalized treatment strategies enhance efficacy by considering immune cell dynamics and patient-specific factors.

## Introduction

Hepatocellular carcinoma (HCC) is the most common type of primary liver cancer, accounting for 80-90% of liver cancer cases (1, 2). It poses a significant global health burden, ranking as the sixth most common cancer and the fourth leading cause of cancer-related deaths, with hundreds of thousands of new cases and related deaths annually (3). Many HCC cases are diagnosed at an advanced stage, at which curative cancer treatment options are no longer viable, and the complexity of the disease limits the effectiveness of surgical interventions (4, 5). Therefore, there is an urgent need for effective therapeutic strategies for advanced HCC.

Transvascular antitumor therapy, commonly including transarterial chemoembolization (TACE), transarterial embolization (TAE), and hepatic arterial infusion chemotherapy (HAIC), has become essential for HCC management, especially for non-surgical candidates (6–8). Interventional therapy has demonstrated strong disease control capabilities and adaptability in the treatment of HCC, leading to its widespread clinical application (9–12).

Immune cell dynamics play a critical role in shaping therapeutic responses and prognosis in HCC patients (13–15). The HCC tumor microenvironment consists of immune cells like macrophages, dendritic cells, T lymphocytes, and natural killer cells, influencing tumor progression and treatment outcomes (11, 16–18). Changes in immune cell composition and activation following interventional therapy significantly impact prognosis. An increase in lymphocytes and a favorable immune phenotype correlate with improved survival and reduced recurrence in cancer patients (19–22). Conversely, an immunosuppressive environment with regulatory T cells and myeloid-derived suppressor cells can contribute to treatment resistance.

Understanding the interplay between interventional therapies and immune response modulation is crucial for optimizing HCC treatment strategies, potentially combining interventional therapies with immune system modulation to enhance anti-tumor responses and overcome resistance (23). Further research into the immune landscape of HCC and its modulation by interventional treatments holds promise for advancing liver cancer therapy.

The dynamic changes in peripheral blood lymphocyte subsets have been recognized as key factors influencing the effectiveness of tumor treatment. However, the potential relationship between the proportions of peripheral lymphocyte subsets and the prognosis of HCC remains unclear. This study focuses on treatment-naïve HCC patients undergoing interventional therapy. It observes changes in peripheral blood leukocytes, particularly lymphocyte subsets, both before treatment and within 28 days after the first interventional therapy. The aim is to explore the potential prognostic value of these changes in predicting the disease control rate and overall effectiveness of interventional therapy in HCC.

## Method

### Participants

**Figure 1:**
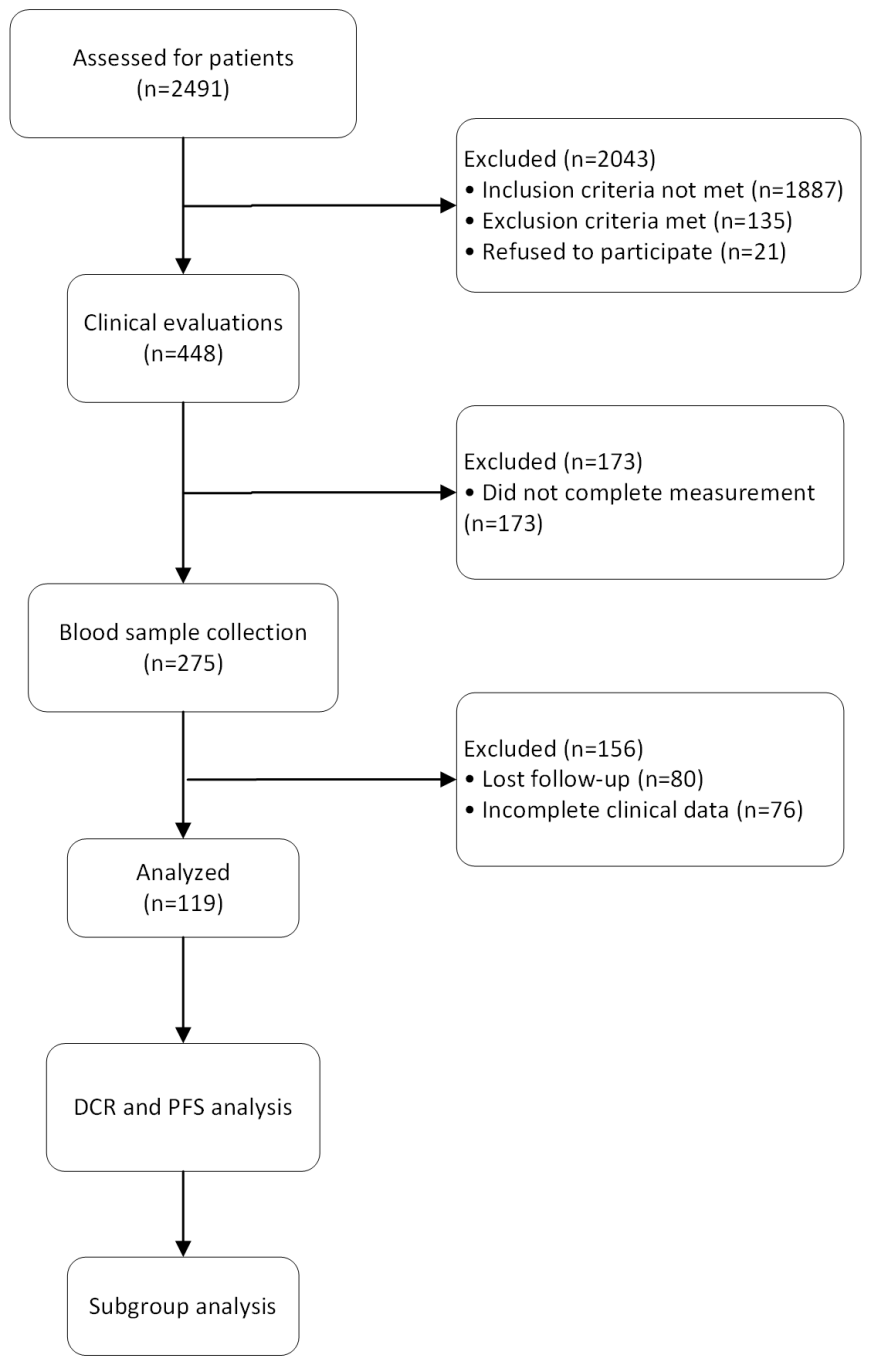
The flow diagram shows the process of the study. 2,491 patients were assessed in the study and 2,043 were excluded due to not meeting eligibility criteria or declining participation. Clinical evaluations were performed on 448 patients, with 173 being excluded due to incomplete measurements. Blood samples were collected from 275 patients, with 156 excluded due to loss to follow-up or incomplete clinical data. The data from 119 patients were used to assess disease control rate and progression-free survival. Subgroup analyses were conducted to explore treatment responses across different patient groups.

This is a single-center observational case-control study, focusing on patients with primary hepatocellular carcinoma admitted to our hospital. The study was approved and overseen by our institution’s ethics committee (based on the Declaration of Helsinki (2021), Ethical Batch No:2020004088), and written informed consent was obtained from the patients or their legal representatives before inclusion. The overall workflow of this study was shown in **Figure 1**.

The criteria for patient inclusion in this study are as follows: adults; pathologically and/or radiologically confirmed primary HCC; no prior antitumor treatment other than surgical resection of HCC; received a comprehensive medical examination at our institution and were deemed suitable for interventional therapy for HCC by a multidisciplinary expert consultation; underwent treatment at our institution and completed follow-up examinations at the designated times; possessed complete clinical data; voluntarily consented to blood lymphocyte subset analysis and sample retention.

The exclusion criteria for patients in this study are as follows: patients who do not consent to participate; patients diagnosed with other tumors or immune system diseases in addition to HCC; patients who did not follow up at the scheduled times or were lost to follow-up; patients with incomplete clinical data; patients deemed unsuitable for further interventional treatment for liver cancer after expert consultation; participation in other clinical trials; severe adverse reactions during anti-tumor treatment; death due to non-tumor-related causes; withdrawal request by the patient or their family; and pregnant or breastfeeding women.

### Treatment process

After confirming the patients’ eligibility for HCC interventional treatment, the procedures were carried out by two interventional radiologists, each with over five years of clinical experience. These radiologists performed the interventions under the guidance of digital subtraction angiography (DSA), ensuring precise and accurate execution. The choice of specific interventional treatment methods was tailored to each patient and was determined based on a comprehensive evaluation of their medical examination results, genetic testing outcomes, personal preferences, and expert consultations.

The selection of the specific interventional therapy (TACE, TAE, or HAIC) was tailored to each patient based on a multidisciplinary evaluation that considered clinical parameters such as tumor size, vascular invasion, liver function, and patient comorbidities.

For TACE, chemotherapeutic agents such as doxorubicin (50 mg/m^2^) mixed with lipiodol (10 mL) were infused into the hepatic artery supplying the tumor, followed by embolization using gel foam or microspheres. Once the Lipiodol emulsion is infused into the hepatic artery, embolic agents such as gelatin sponge particles, drug-eluting beads, or polyvinyl alcohol particles are delivered to block the blood flow, trapping the chemotherapeutic agent within the tumor and leading to ischemia.

The TAE procedure was guided by DSA to ensure precise localization of the tumor and effective embolization of the feeding vessels. In TAE treatment, the embolization of the tumor’s blood supply was achieved by injecting embolic agents such as microspheres (100–300 µm) or gelfoam particles under DSA guidance. The choice of embolic material was based on the tumor’s size, vascularity, and proximity to critical structures. Embolic materials such as gelatin sponge particles, polyvinyl alcohol particles, or calibrated microspheres, typically ranging in size from 100 to 500 microns, are injected to block the tumor’s blood supply.

In HAIC, the FOLFOX regimen typically involves the intra-arterial administration of oxaliplatin, 5-fluorouracil (5-FU), and leucovorin through a catheter placed in the hepatic artery. Oxaliplatin is usually administered first as a 2-hour infusion at a dose of 85 mg/m². This is followed by leucovorin, administered over 2 hours at a dose of 400 mg/m², which enhances the effect of 5-FU. After leucovorin, 5-FU is given as a bolus of 400 mg/m², followed by a continuous infusion of 2400 mg/m² over 46 hours. This regimen is typically repeated every 2 weeks.

Following the interventional treatment, patients were closely monitored and within 28 days, they underwent a follow-up imaging procedure to assess the treatment’s effectiveness. This follow-up imaging involved either an enhanced computed tomography (CT) scan or magnetic resonance imaging (MRI) scan of the liver, providing detailed visual information on the hepatic condition post-treatment. These imaging techniques were crucial for evaluating the response to the intervention and planning any further necessary treatments.

Following transvascular interventional therapies, most patients achieved satisfactory disease control based on post-treatment imaging and clinical evaluations. Given the effective control of the lesions observed in these patients, as well as personal preferences, patients in this study opted not to undergo additional antitumor therapies such as systemic targeted therapy, immunotherapy, or further local ablative treatments. The decision to forgo additional treatment was made in consultation with the clinical team and was based on the individual patient’s disease status, response to initial therapy, and personal treatment preferences.

### Sample collection

Venous blood samples were collected from patients in the morning after an overnight fast to ensure consistency and accuracy of the biochemical measurements. After collection, the blood samples were immediately processed. Whole blood was used for routine blood tests and tumor marker analysis. The blood samples were then subjected to centrifugation at 3000 rpm for 10 minutes to separate the serum, which was subsequently aliquoted and stored at -80°C until further analysis. The complete blood count was performed using a hematology analyzer, which measured parameters such as WBCs count, red blood cell count, hemoglobin concentration, hematocrit, and platelet count. Tumor markers, including alpha-fetoprotein (AFP) and others relevant to hepatocellular carcinoma, were quantitatively assessed using specific immunoassay techniques on the automated analyzer.

B cells (CD3 − CD19+), total T cells (CD3 +), natural killer cells (NK cells, CD3-/CD16 + CD56+), helper T cells (Th cells, CD3 + CD4+), and cytotoxic T cells/suppressor T cells (Tc/Ts cells, CD3 + CD8+) were identified and quantified by flow cytometry. The percentages of lymphocyte subsets among total white blood cells were calculated.

### Follow-up and outcomes

Our institution is responsible for the initial treatment, diagnosis, multidisciplinary expert consultations, interventional therapy, and the entire follow-up period of the patients to ensure close monitoring and timely intervention when necessary. Following the initial interventional treatment and until the lesions are confirmed to be completely controlled, patients undergo comprehensive evaluations every 28 days (24). These evaluations include repeated laboratory tests, enhanced imaging assessments, and clinical evaluations. Laboratory tests comprise routine blood tests and tumor marker analysis, conducted using the same standardized procedures and automated biochemical analyzers described in the initial sample collection and analysis protocol.

Enhanced imaging assessments are performed using CT/MRI to monitor the lesions in the upper abdominal liver region and detect any changes in the patient’s condition. Each follow-up visit also involves thorough clinical evaluations, including physical examinations and symptom reviews. The data collected from laboratory tests, imaging studies, and clinical evaluations are meticulously recorded and integrated into the patients’ medical records. Data collection is independently conducted by two researchers, and any discrepancies are arbitrated by the project leader and the research team lead.

Progression-free survival (PFS) was defined as the duration of time between treatment completion and clinical disease progression. The primary outcomes of this study were defined as disease control rate (DCR), which includes complete response (CR), partial response (PR), and stable disease (SD) according to the modified RECIST (mRECIST) criteria (25). Secondary outcomes included changes in immune cell dynamics, particularly total white blood cell count, lymphocyte count, monocyte count, and basophil count before and after interventional therapy. Additional secondary outcomes included associations between clinical variables (BMI, diabetes, infection, multiple lesions) and immune cell abnormalities, such as T cell and B cell dynamics. Conversely, patients who experience disease progression are deemed to have failed tumor control.

### Statistical analysis

All data were statistically analyzed using R software (R software, version 4.2.0; https://www.r-project.org/about.html), and GraphPad (GraphPad Prism 9; GraphPad Software, Inc.) was utilized for graphical representations. Categorical variables are expressed as frequencies, and comparisons were made using the chi-square test. Continuous data following a normal distribution are presented as mean ± standard deviation or median (interquartile range), with comparisons conducted using t-tests or one-way ANOVA. All tests were considered statistically significant at *P*<0.05.

## Results

### Participants

From Jun. 1, 2021, to April 1, 2024, a total of 2,491 HCC patients received transvascular antitumor interventional therapies at our institution. Out of these, 448 patients underwent specific interventional procedures. After screening, 275 patients met the inclusion criteria for the study, and 119 patients with complete data were ultimately included. The study population consisted of 102 males and 17 females, with a mean age of 59.26 ± 9.86 years. Among the patients, 53 received TACE alone, 46 received TAE alone, 11 received a combination of TACE and HAIC, and 9 received a combination of TAE and HAIC. Detailed clinical baseline data are provided in **Table 1**.

**Table 1:** Clinical baseline characteristics of patients.

Variables, n (%)	Total (n = 119)	Failure Control Group (n = 62)	Success Control Group (n = 57)	Statistic	*P*
Age, Mean ± SD	59.26 ± 9.86	59.73 ± 9.66	58.75 ± 10.14	t=0.54	0.594
BMI, Mean ± SD	24.03 ± 3.53	23.71 ± 3.23	24.38 ± 3.83	t=-1.03	0.306
AFP, Mean ± SD	7856.31 ± 16862.88	8539.44 ± 16834.97	7113.26 ± 17011.19	t=0.46	0.647
AST, Mean ± SD	67.23 ± 57.45	74.76 ± 63.41	59.04 ± 49.45	t=1.50	0.137
ALT, Mean ± SD	50.07 ± 39.75	52.09 ± 43.61	47.89 ± 35.34	t=0.57	0.567
S/L, Mean ± SD	1.59 ± 0.98	1.65 ± 1.10	1.52 ± 0.83	t=0.69	0.490
Gender				χ²=0.36	0.549
Male	102 (85.71)	52 (83.87)	50 (87.72)		
Female	17 (14.29)	10 (16.13)	7 (12.28)		
Age group				χ²=1.52	0.218
<60 years	64 (53.78)	30 (48.39)	34 (59.65)		
≥60 years	55 (46.22)	32 (51.61)	23 (40.35)		
Alcohol abuse				χ²=1.25	0.264
No	73 (61.34)	41 (66.13)	32 (56.14)		
Yes	46 (38.66)	21 (33.87)	25 (43.86)		
Smoking				χ²=0.33	0.569
No	70 (58.82)	38 (61.29)	32 (56.14)		
Yes	49 (41.18)	24 (38.71)	25 (43.86)		
Family cancer history				χ²=0.01	0.940
No	102 (85.71)	53 (85.48)	49 (85.96)		
Yes	17 (14.29)	9 (14.52)	8 (14.04)		
Weight loss				χ²=2.03	0.154
No	89 (74.79)	43 (69.35)	46 (80.70)		
Yes	30 (25.21)	19 (30.65)	11 (19.30)		
BMI abnormal				χ²=1.98	0.160
No	56 (47.06)	33 (53.23)	23 (40.35)		
Yes	63 (52.94)	29 (46.77)	34 (59.65)		
Diabetes				χ²=1.20	0.273
No	108 (90.76)	58 (93.55)	50 (87.72)		
Yes	11 (9.24)	4 (6.45)	7 (12.28)		
Hypertension				χ²=0.75	0.386
No	81 (68.07)	40 (64.52)	41 (71.93)		
Yes	38 (31.93)	22 (35.48)	16 (28.07)		
PVTT				χ²=0.01	0.937
No	81 (68.07)	42 (67.74)	39 (68.42)		
Yes	38 (31.93)	20 (32.26)	18 (31.58)		
PH				χ²=0.37	0.541
No	91 (76.47)	46 (74.19)	45 (78.95)		
Yes	28 (23.53)	16 (25.81)	12 (21.05)		
Infection				χ²=1.58	0.209
No	103 (86.55)	56 (90.32)	47 (82.46)		
Yes	16 (13.45)	6 (9.68)	10 (17.54)		
Distant metastasis				χ²=1.58	0.209
No	103 (86.55)	56 (90.32)	47 (82.46)		
Yes	16 (13.45)	6 (9.68)	10 (17.54)		
Multiple lesions				χ²=0.16	0.686
No	67 (56.30)	36 (58.06)	31 (54.39)		
Yes	52 (43.70)	26 (41.94)	26 (45.61)		
Maximum diameter over 50mm				χ²=0.03	0.874
No	51 (42.86)	27 (43.55)	24 (42.11)		
Yes	68 (57.14)	35 (56.45)	33 (57.89)		
Lymph nodes, n(%)				χ²=0.51	0.477
No	84 (70.59)	42 (67.74)	42 (73.68)		
Yes	35 (29.41)	20 (32.26)	15 (26.32)		
Ascites				χ²=4.11	0.043
No	88 (73.95)	41 (66.13)	47 (82.46)		
Yes	31 (26.05)	21 (33.87)	10 (17.54)		
Splenomegaly				χ²=0.17	0.683
No	75 (63.03)	38 (61.29)	37 (64.91)		
Yes	44 (36.97)	24 (38.71)	20 (35.09)		
CNCL stage				χ²=0.93	0.335
I-II	53 (44.54)	25 (40.32)	28 (49.12)		
III-IV	66 (55.46)	37 (59.68)	29 (50.88)		
Liver Cirrhosis				χ²=0.74	0.388
No	64 (53.78)	31 (50.00)	33 (57.89)		
Yes	55 (46.22)	31 (50.00)	24 (42.11)		
Hepatitis				χ²=0.11	0.744
No	40 (33.61)	20 (32.26)	20 (35.09)		
Yes	79 (66.39)	42 (67.74)	37 (64.91)		
DCR				_	_
CR		_	3 (5.26)		
PR		_	37 (64.92)		
SD^†^		_	17 (29.82)		

BMI, Body Mass Index; AFP, Alpha-fetoprotein; AST, Aspartate Aminotransferase; ALT, Alanine Aminotransferase; S/L, AST/ALT; PVTT, Portal Vein Tumor Thrombus; PH, Portal Hypertension; CNCL stage, China Liver Cancer Stage; SD, standard deviation; DCR, disease control rate; CR, complete response; PR, partial response; SD^†^, stable disease; t, t-test, χ², Chi-square test.

### Overall WBCs analysis

**Figure 2:**
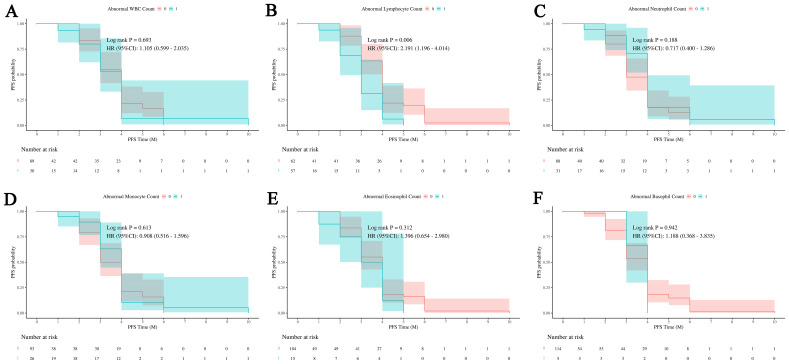
Impact of white blood cell subsets on PFS in HCC. Survival analysis shows the relationship between progression-free survival (PFS) and abnormal counts across various white blood cell (WBC) subsets. **(A)** The relationship between abnormal WBC counts and PFS outcomes in patients. **(B)** The relationship between abnormal lymphocyte count and PFS outcomes in patients. **(C)** The relationship between abnormal neutrophil count and PFS outcomes in patients. **(D)** The relationship between abnormal monocyte count and PFS outcomes in patients. **(E)** The relationship between abnormal eosinophil count and PFS outcomes in patients. **(F)** The relationship between abnormal basophil count and PFS outcomes in patients. Abnormal WBC, neutrophil, monocyte, eosinophil, and basophil counts demonstrate no significant effect on PFS, while abnormal lymphocyte counts are associated with an increased risk of disease progression. 0: No abnormality in cell count or proportion; 1: Abnormality in cell count or proportion.

**Figure 2** shows the probability of PFS in patients with overall WBCs counts and with normal and abnormal counts in different WBCs subsets. **Figure 2A** indicates that abnormal WBCs count has no significant impact on PFS, with a log-rank test *P* -value of 0.693 and a hazard ratio (HR) of 1.105 (95% confidence interval [CI] of 0.599 to 2.035). **Figure 2B** shows that abnormal lymphocyte count has a significant impact on PFS, with a log-rank test *P* -value of 0.006 and a hazard ratio of 2.191 (95% CI of 1.196 to 4.014), indicating that patients in abnormal group have 2.191 times the risk of disease progression compared to normal group. **Figure 2C** indicates that abnormal neutrophil count has no significant impact on PFS, with a log-rank test *P* -value of 0.188 and a hazard ratio of 0.717 (95% CI of 0.400 to 1.286). **Figure 2D** shows that abnormal monocyte count has no significant impact on PFS, with a log-rank test *P*-value of 0.613 and a hazard ratio of 0.908 (95% CI of 0.516 to 1.596). **Figures 2E**, **F** indicate that abnormal eosinophil and basophil counts have no significant impact on PFS, with log-rank test *P* -values of 0.312 and 0.942, and hazard ratios of 1.396 (95% CI of 0.654 to 2.980) and 1.188 (95% CI of 0.368 to 3.835), respectively.

**Figure 3:**
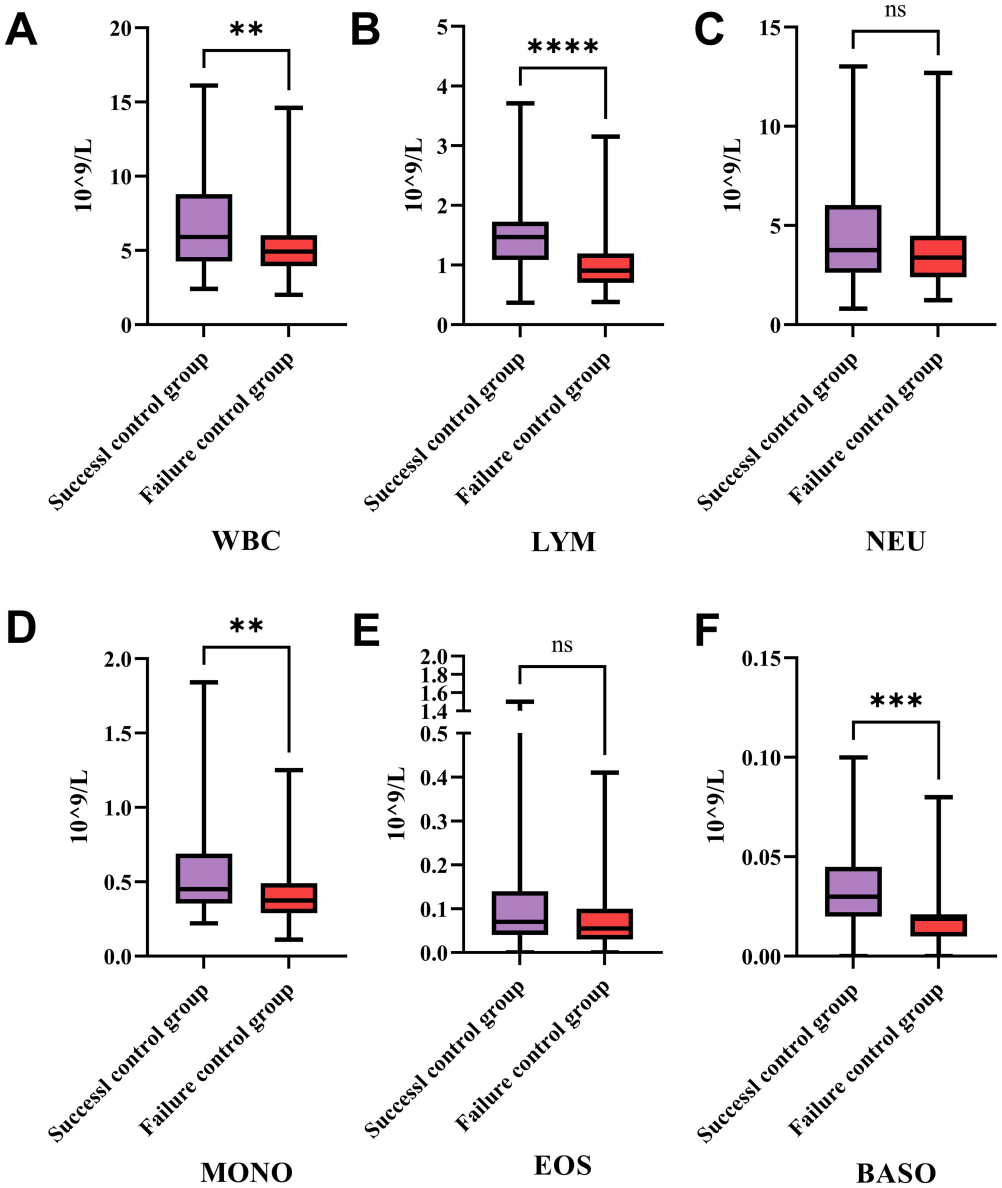
Differences in white blood cell subtypes between success and failure control groups in treatment. This figure compares the counts of various WBC types between the success and failure control groups. **(A)** The expression levels of WBC in HCC patients from the success and failure control groups after therapies. **(B)** The expression levels of lymphocyte in HCC patients from the success and failure control groups after therapies. **(C)** The expression levels of neutrophil in HCC patients from the success and failure control groups after therapies. **(D)** The expression levels of monocyte in HCC patients from the success and failure control groups after therapies. **(E)** The expression levels of eosinophil in HCC patients from the success and failure control groups after therapies. **(F)** The expression levels of basophil in HCC patients from the success and failure control groups after therapies. Patients with successful treatment show significantly higher total WBC, lymphocyte, monocyte, and basophil counts compared to those with failed treatment, indicating potential correlations between these cell types and treatment outcomes. No significant differences are observed in neutrophil and eosinophil counts between the two groups, suggesting these cell types may not play a major role in influencing treatment success. WBC, White Blood Cells; LYM, Lymphocytes; NEU, Neutrophils; MONO, Monocytes; EOS, Eosinophils; BASO, Basophils; *P < 0.05, ** *P* < 0.01, *** *P* < 0.001, **** *P* < 0.0001; ns: no statistically significant difference.

The **Figure 3** illustrates the differences in the counts of various types of white blood cells between the success control group and the failure control group. **Figure 3A** shows that the total white blood cell count in the success control group is significantly higher than that in the failure control group, with a *P* -value less than 0.01. This suggests that in patients with successful treatment, the total white blood cell count is significantly higher than in those with failed treatment, potentially indicating a correlation between the total white blood cell count and treatment outcomes.

**Figure 3B** indicates that the lymphocyte count in the success control group is significantly higher than in the failure control group, with a *P* -value less than 0.0001. This implies that in patients with successful treatment, the number of lymphocytes is significantly higher than in those with failed treatment, suggesting an important role of lymphocytes in treatment success. **Figure 3C** shows no significant difference in neutrophil counts between the two groups, indicating that neutrophil count does not significantly differ between the success and failure control groups, and may not be a key factor influencing treatment outcomes. **Figure 3D** shows that the monocyte count in the success control group is significantly higher than in the failure control group, with a *P* -value less than 0.01. This suggests that in patients with successful treatment, the number of monocytes is significantly higher than in those with failed treatment, indicating that monocytes may play a role in treatment success. **Figure 3E** indicates no significant difference in eosinophil counts between the two groups, suggesting that eosinophil count does not significantly differ between the success and failure control groups, and may not be a key factor influencing treatment outcomes. **Figure 3F** shows that the basophil count in the success control group is significantly higher than in the failure control group, with a *P* -value less than 0.001.

### Lymphatic subgroup analysis

**Figure 4:**
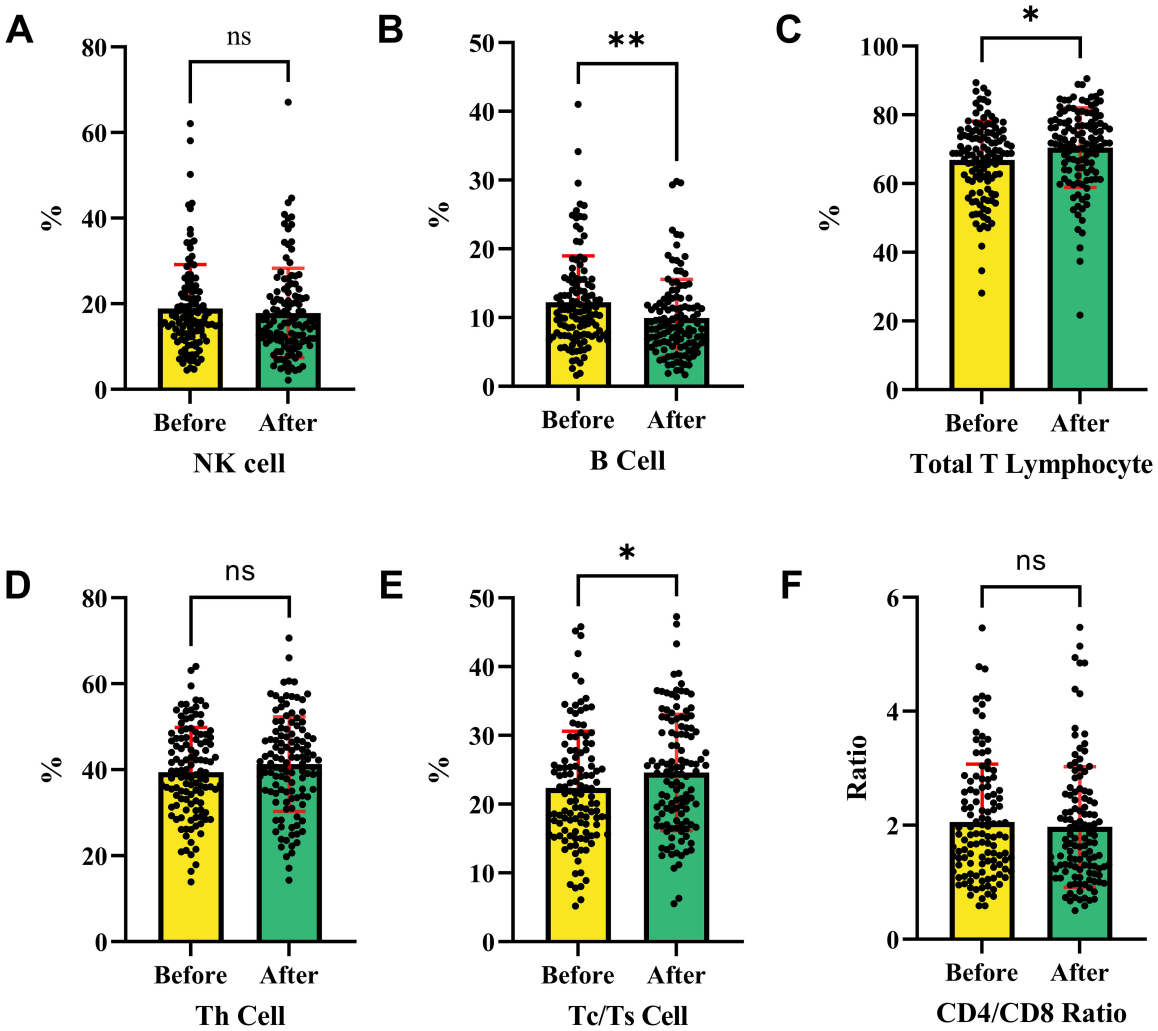
Changes in immune cell subpopulations before and after interventional therapy in HCC. This analysis shows the changes in immune cell populations before and after therapies. **(A)** The ratio of NK cell in HCC patients before and after therapies. **(B)** The ratio of B cell in HCC patients before and after therapies. **(C)** The ratio of total T lymphocytes in HCC patients before and after therapies. **(D)** The ratio of Th cell in HCC patients before and after therapies. **(E)** The ratio of Tc/Ts cell in HCC patients before and after therapies. **(F)** The ratio of CD4/CD8 in HCC patients before and after therapies. NK cells and helper T cells remained stable with no significant change, while B cells, total T lymphocytes, and Tc/Ts cells increased post-treatment, indicating activation of humoral and cytotoxic immune responses. The CD4/CD8 ratio showed no significant variation, suggesting that the intervention had no effect on this ratio. NK cells, Natural Killer cells; Th cells, Helper T cells (T helper cells); Tc/Ts cells, Cytotoxic T cells/Suppressor T cells; *P < 0.05, ** *P* < 0.01, *** *P* < 0.001, **** *P* < 0.0001; ns, no statistically significant difference.

**Figure 4** shows changes in immune cell percentages or ratios before and after intervention treatment. **Figure 4A** indicates no significant change in NK cells, suggesting no impact on their proportion. **Figure 4B** shows a significant increase in B cells post-treatment, indicating a potential activation of the humoral immune response. **Figure 4C** reveals a significant increase in total T lymphocytes, suggesting an enhanced T cell-mediated response.

**Figure 4D** indicates no significant change in Th cells, suggesting their stability. **Figure 4E** demonstrates a significant increase in Tc/Ts cells, suggesting an enhanced cytotoxic immune response. **Figure 4F** shows no significant change in the CD4/CD8 ratio, indicating no effect on this ratio. Overall, the intervention significantly increased B cells, total T lymphocytes, and Tc/Ts cells, indicating potential activation of humoral and cytotoxic immune responses. However, NK cells, helper T cells, and the CD4/CD8 ratio remained stable before and after treatment.

**Figure 5:**
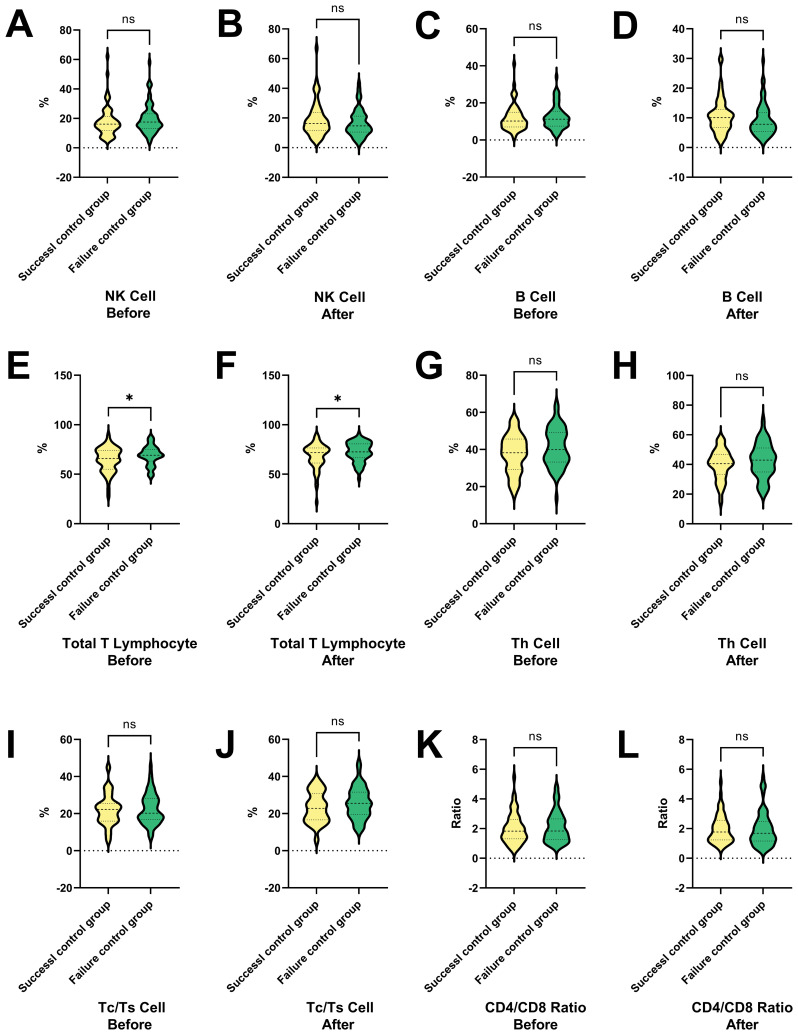
Changes in T cell subtypes and ratios in success and failure control groups before and after interventional therapy in HCC. This figure presents the percentage changes in various T cell types and their ratios between the success and failure control groups before and after treatment. **(A, B)** The ratio of NK cell in HCC patients in the success and failure control groups before and after therapies. **(C, D)** The ratio of B cell in HCC patients in the success and failure control groups before and after therapies. **(E, F)** The ratio of total T lymphocytes in HCC patients in the success and failure control groups before and after therapies. **(G, H)** The ratio of Th cell in HCC patients in the success and failure control groups before and after therapies. **(I, J)** The ratio of Tc/Ts cell in HCC patients in the success and failure control groups before and after therapies. **(K, L)** The ratio of CD4/CD8 in HCC patients in the success and failure control groups before and after therapies. NK cells and B cells show no significant differences between the groups, indicating that they do not impact treatment outcomes. In contrast, total T lymphocytes are significantly higher in the success group both before and after treatment, suggesting their key role in treatment success. Other immune cell populations, including Th cells, Tc/Ts cells, and the CD4/CD8 ratio, remain stable and do not significantly affect outcomes. NK cell, Natural Killer cells; Th cell, Helper T cell (T helper cells); Tc/Ts cell, Cytotoxic T cells/Suppressor T cells; *P < 0.05; ns, no statistically significant difference.

**Figure 5** shows the percentage changes of various T cell types and their ratios between the success and failure control groups before and after treatment. **Figures 5A**, **B** show no significant differences in NK cells between the groups, suggesting NK cells do not impact treatment outcomes, while **Figures 5C**, **D** indicate no significant differences in B cells, implying B cells remain stable and do not affect outcomes. **Figure 5E** shows a significantly higher percentage of total T lymphocytes in the success group before treatment (*P* < 0.05), suggesting their importance in treatment success. **Figure 5F** confirms this higher percentage after treatment, further supporting their role. **Figures 5G–L** show no significant differences in Th cells, Tc/Ts cells, and the CD4/CD8 ratio, indicating that these immune cell populations remain stable and do not impact outcomes. Total T lymphocytes are significantly higher in the success group before and after treatment, suggesting their key role in treatment success, while NK cells, B cells, Th cells, Tc/Ts cells, and the CD4/CD8 ratio are not significant factors. Further research is needed to clarify the mechanisms and clinical significance of total T lymphocytes in treatment success.

**Figure 6:**
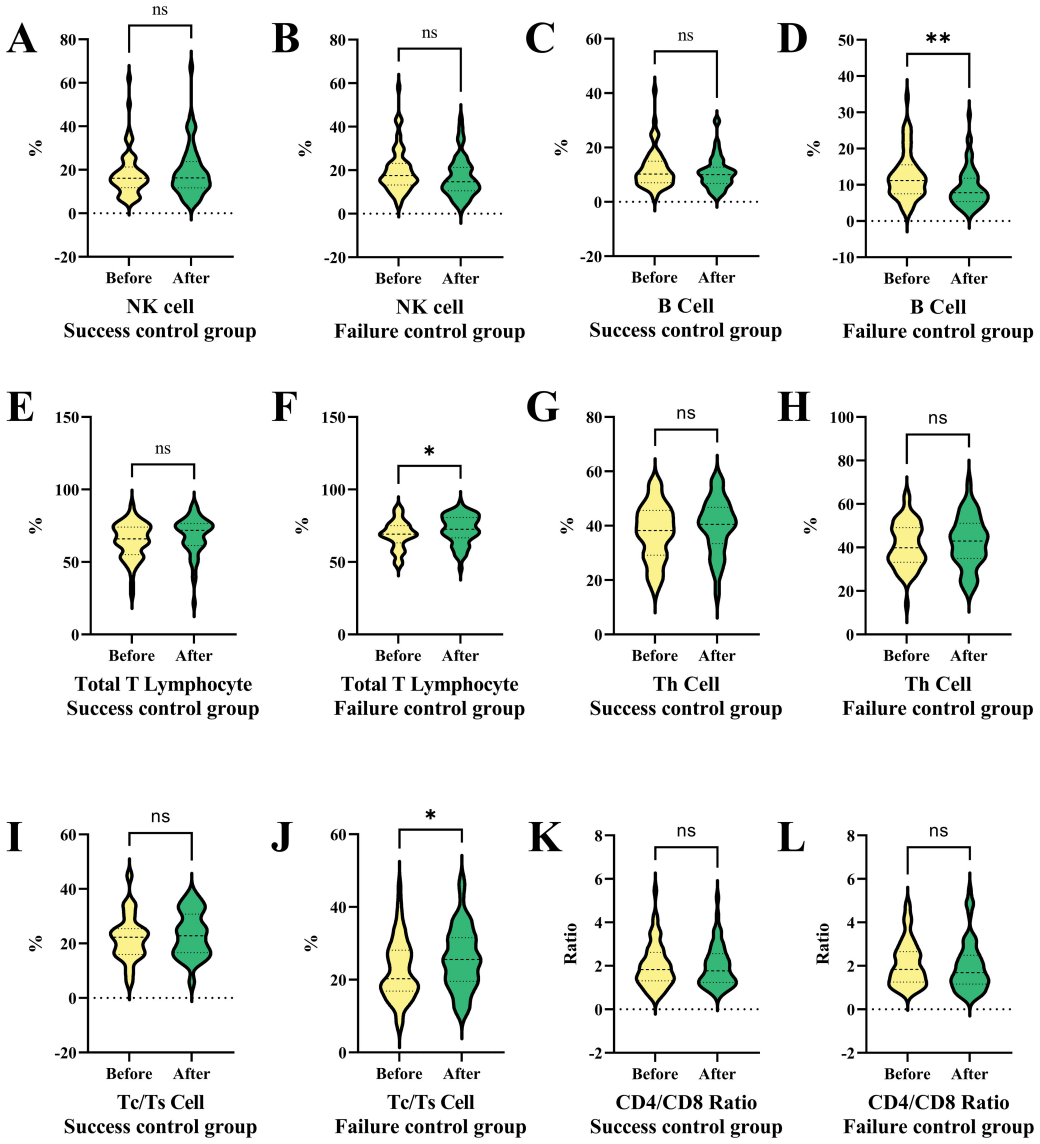
Comparative analysis of immune cell populations and ratios in success and failure control groups before and after interventional therapy in HCC. This figure compares the changes in immune cell populations and ratios before and after treatment in both success and failure control groups using violin plots. **(A)** NK cell ratio in success subgroup before and after therapy. **(B)** NK cell ratio in failure subgroup before and after therapy. **(C)** B cell ratio in success subgroup before and after therapy. **(D)** B cell ratio in failure subgroup before and after therapy. **(E)** Total T lymphocyte ratio in success subgroup before and after therapy. **(F)** Total T lymphocyte ratio in failure subgroup before and after therapy. NK cells and Th cells show no significant differences in either group, while B cells increase significantly only in the failure group. Total T lymphocytes and Tc/Ts cells remain stable in the success group but increase notably in the failure group, indicating potential immune dysregulation associated with tumor progression. CD4/CD8 ratios show no significant changes in either group. NK cells, Natural Killer cells; Th cells, Helper T cells (T helper cells); Tc/Ts cells, Cytotoxic T cells/Suppressor T cells; *P < 0.05, ** *P* < 0.01; ns, no statistically significant difference.

**Figure 6** presents a series of violin plots comparing the changes in various immune cell populations and ratios before and after a specific treatment or condition in both success and failure control groups. The results show that NK cells and Th cells exhibit no significant differences in both control groups before and after the treatment. B cells show no significant difference in the success group but significantly increase in the failure group.

Total T lymphocytes and Tc/Ts cells show no significant difference in the success control group but significantly increase in the failure patients (**Figures 6F, J**). CD4/CD8 ratios exhibit no significant differences in both control groups before and after the treatment (**Figure 6K**). The failure control group in **Figure 6** shows significant changes in B cells, total T lymphocytes, and Tc/Ts cells after the treatment or condition, suggesting a specific immune response or dysregulation related to tumor progression. The remaining comparison results (**Figures 6A–E, G–I, L**) did not show significant statistical differences.

### Subgroup analysis

**Figure 7:**
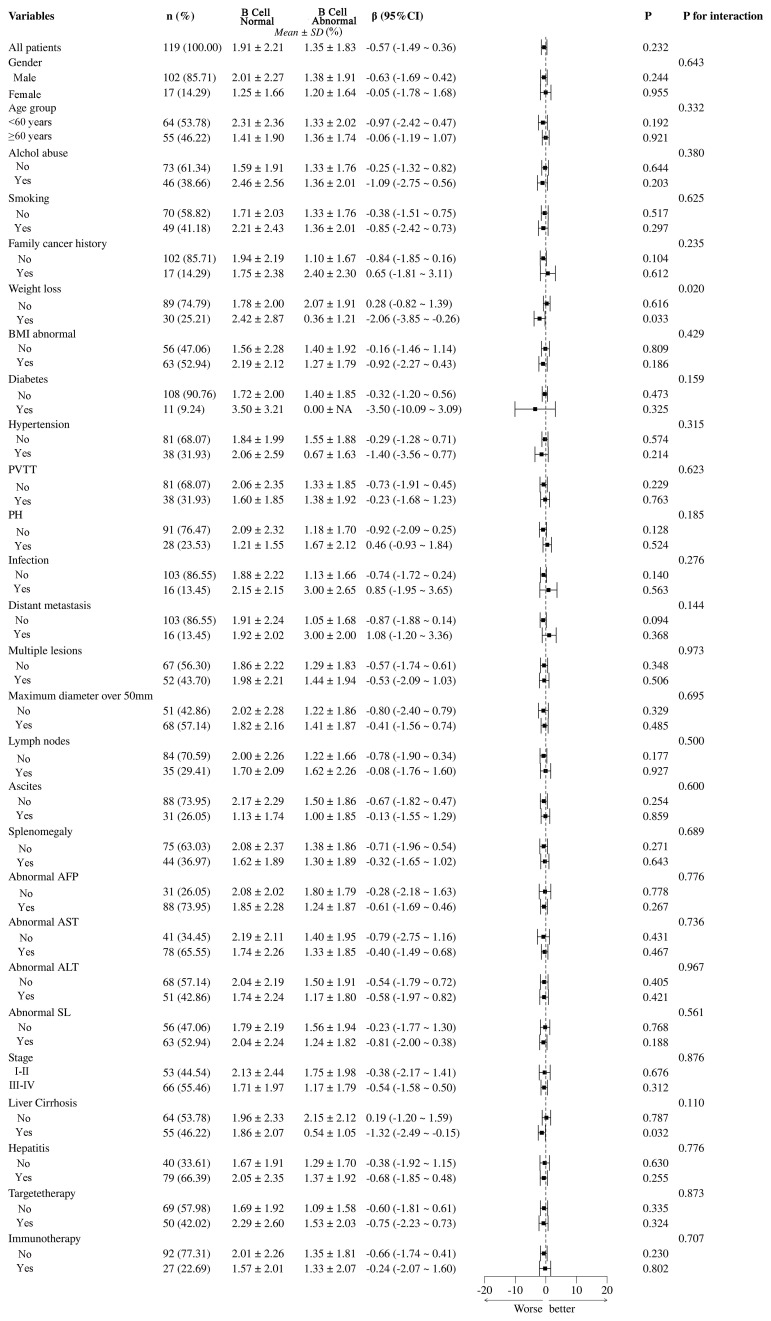
Subgroup analysis of B cell abnormalities and their clinical and pathological impacts in HCC patients. PVTT, portal vein tumor thrombus; PH, portal hypertension; BMI, Body Mass Index; NA, Not Available.

In this analysis shown in **Figure 7**, the differences between B cell normal and abnormal groups were analyzed across 119 patients, highlighting the significant impact of various clinical and pathological variables. Statistical analysis using β coefficients (95% CI) and P-values revealed that weight loss (β = -2.08, 95% CI = -1.82 to -1.39, *P* = 0.020) and abnormal Body Mass Index (BMI) (β = -2.06, 95% CI = -3.85 to -0.26, *P* = 0.429) were significantly lower in the B cell abnormal group. Additionally, portal vein tumor thrombus (PVTT) and multiple lesions also showed significant negative correlations in the B cell abnormal group, with β values of -1.73 (95% CI = -2.91 to -0.45, *P* = 0.229) and -0.57 (95% CI = -1.74 to -0.61, *P* = 0.348), respectively. These results suggest that weight loss, abnormal BMI, PVTT, and multiple lesions may be key factors influencing B cell abnormalities in patients. Other variables, such as gender, age group, smoking, and alcohol abuse, did not show significant differences in relation to B cell abnormalities.

**Figure 8:**
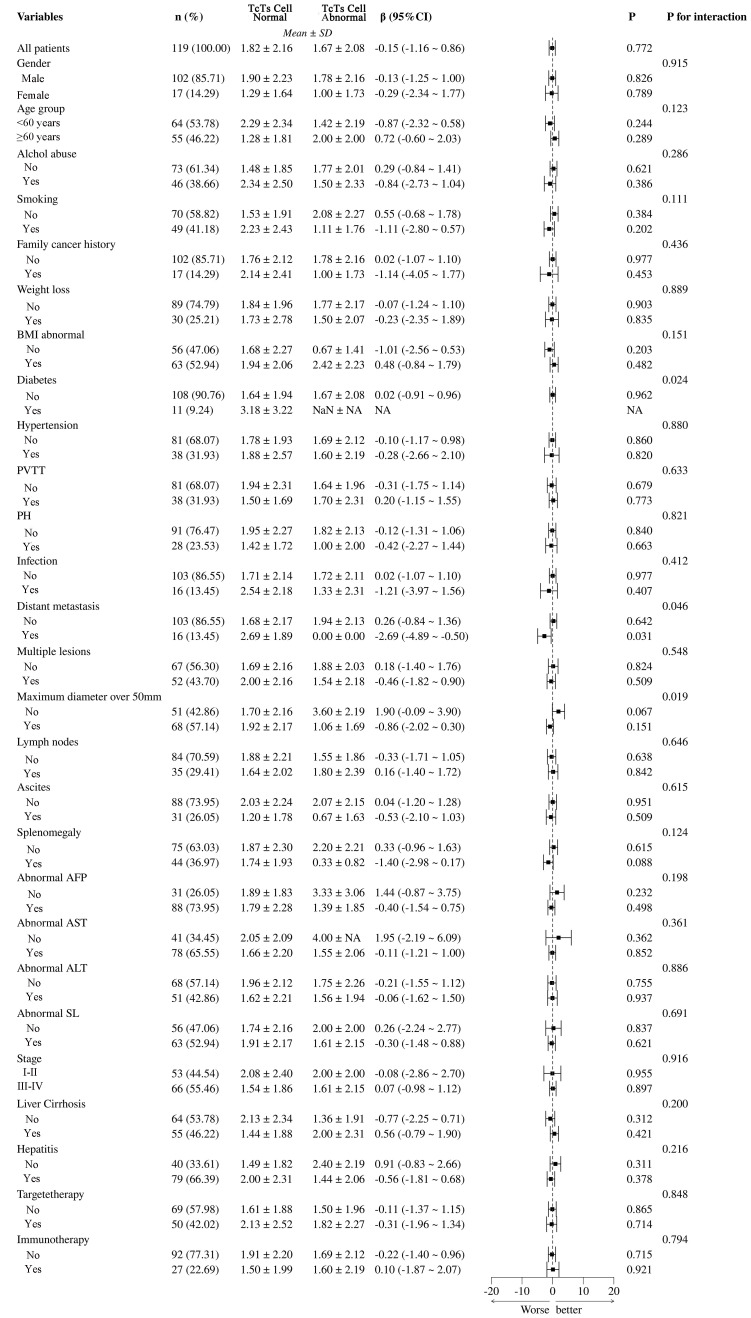
Subgroup analysis of Ts/TC cell abnormalities and their clinical and pathological impacts in HCC patients. PVTT, portal vein tumor thrombus; PH, portal hypertension; BMI, Body Mass Index; NA, Not Available.

The analysis between normal and abnormal CD3+CD8+ cell groups demonstrates that various clinical and pathological variables have significant impacts, as shown in **Figure 8**. Abnormal BMI (β = -1.01, 95% CI = -2.56 to -0.53, *P* = 0.020) and diabetes (β = 0.02, 95% CI = -0.91 to -0.96, *P* = 0.024) are significantly linked to the T cell abnormal group. Infection (β = -0.31, 95% CI = -1.21 to -0.37, *P* = 0.046) and multiple lesions (β = -2.69, 95% CI = -4.89 to -0.50, *P* = 0.031) also show significant negative correlations with T cell abnormalities. These findings suggest that abnormal BMI, diabetes, infection, and multiple lesions are key factors influencing T cell abnormalities in patients. Other variables such as gender, age group, smoking, and alcohol abuse do not show significant differences related to T cell abnormalities.

**Figure 9:**
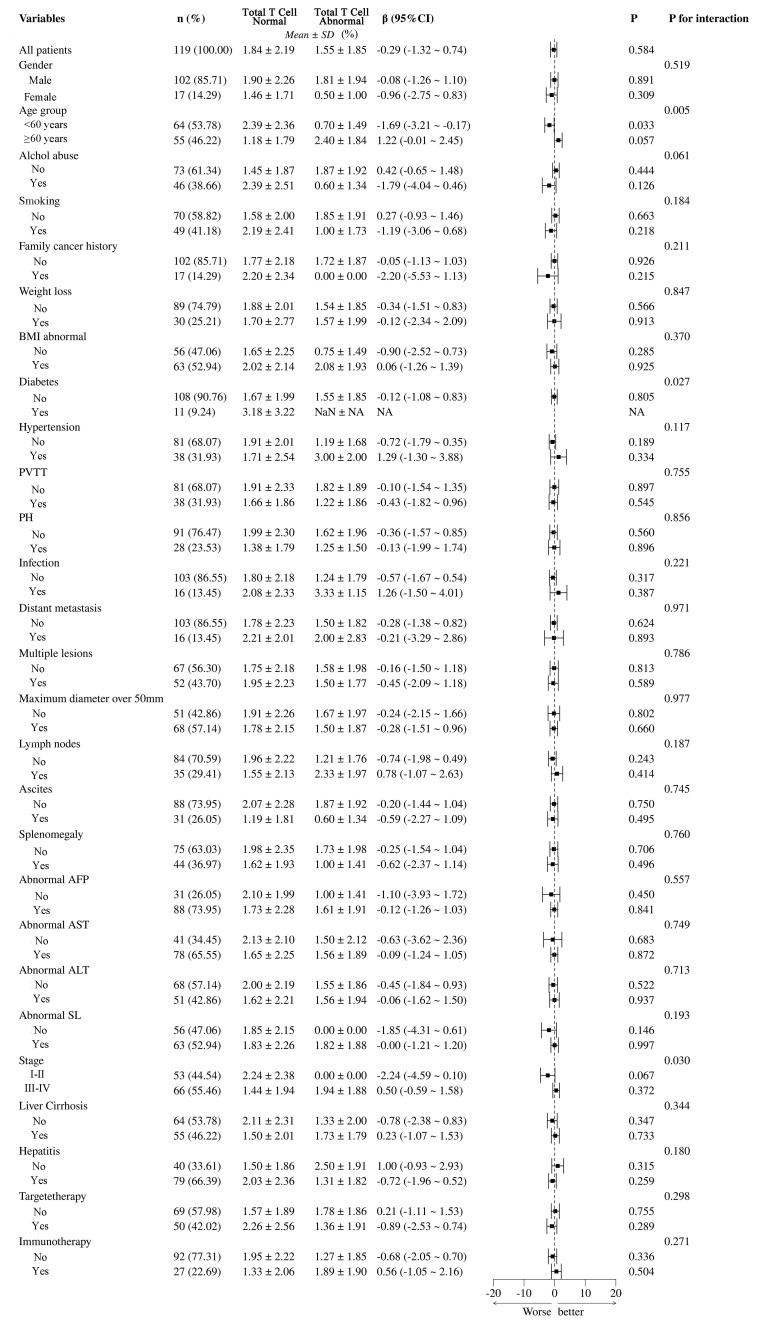
Subgroup analysis of total T cell abnormalities and their clinical and pathological impacts in HCC patients. PVTT, portal vein tumor thrombus; PH, portal hypertension; BMI, Body Mass Index; NA, Not Available.

In **Figure 9**, the differences between total T cell normal and abnormal groups, highlighting the significant impact of various clinical and pathological variables. Statistical analysis using β coefficients (95% CI) and P-values revealed that age group (β = -1.69, 95% CI = -3.21 to -0.17, *P* = 0.033) and abnormal BMI (β = -0.90, 95% CI = -2.52 to -0.73, *P* = 0.027) were significantly lower in the T cell abnormal group. Additionally, hypertension and abnormal AFP also showed significant negative correlations in the T cell abnormal group, with β values of -0.72 (95% CI = -1.79 to -0.35, *P* = 0.026) and -1.10 (95% CI = -2.36 to -1.03, *P* = 0.015), respectively. These results suggest that age group, abnormal BMI, hypertension, and abnormal AFP may be key factors influencing T cell abnormalities in patients. Other variables, such as gender, smoking, and alcohol abuse, did not show significant differences in relation to T cell abnormalities.

**Figure 10:**
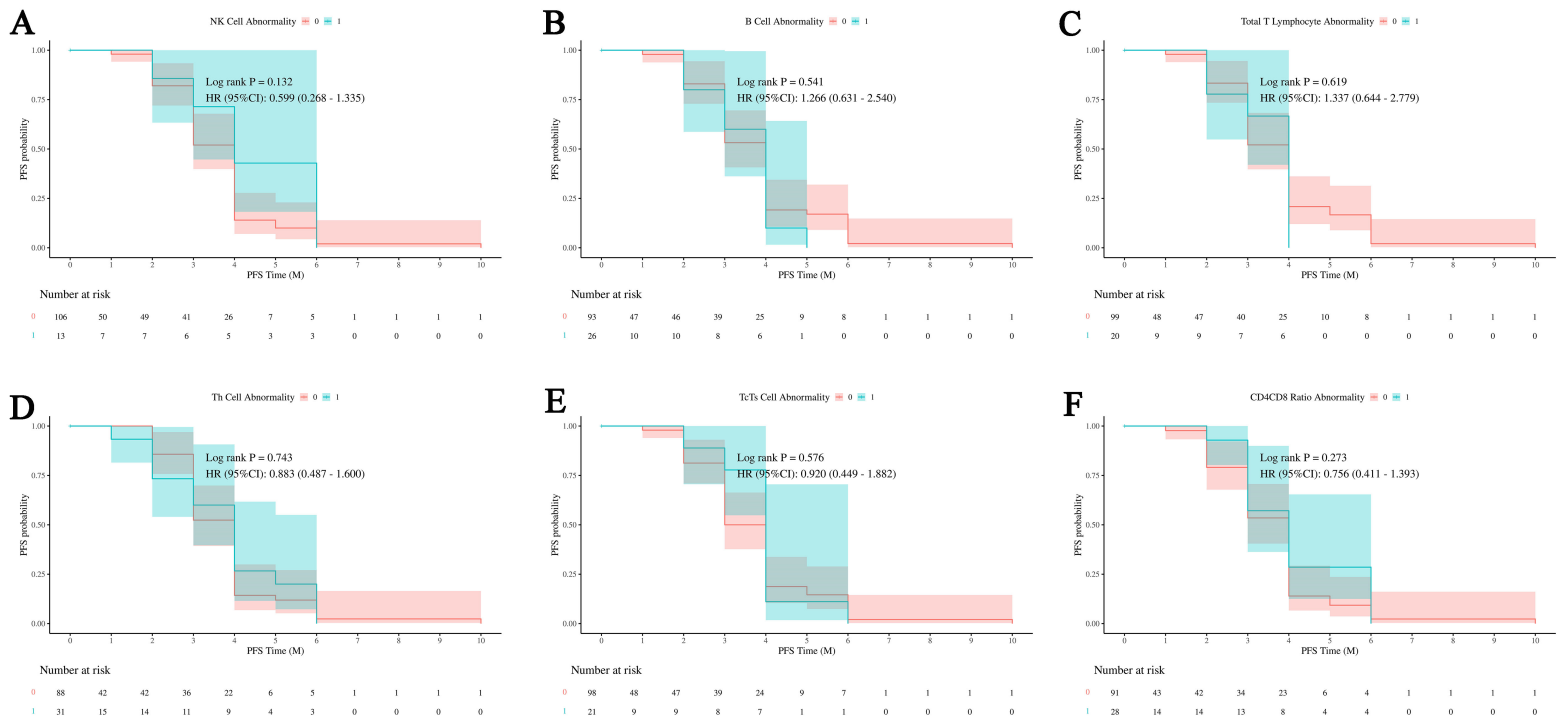
Impact of immune cell abnormalities on PFS in HCC. The relationship between immune cell abnormalities, including NK cells, B cells, total T lymphocytes, Th cells, Tc/Ts cells, and CD4/CD8 ratios, and progression-free survival (PFS). **(A)**The relationship between abnormal NK cell ratio and PFS outcomes in patients. **(B)** The relationship between abnormal B cell ratio and PFS outcomes in patients. **(C)** The relationship between abnormal total T lymphocytes ratio and PFS outcomes in patients. **(D)** The relationship between abnormal Th cell ratio and PFS outcomes in patients. **(E)** The relationship between abnormal Tc/Ts cell and PFS outcomes in patients. **(F)** The relationship between abnormal CD4/CD8 and PFS outcomes in patients. The analysis shows that none of these immune cell abnormalities have a significant impact on PFS, indicating no clear influence on patient outcomes within this cohort. NK cells, Natural Killer cells; Th cells, Helper T cells (T helper cells); Tc/Ts cells, Cytotoxic T cells/Suppressor T cells; 0, No abnormality in cell count or proportion; 1, Abnormality in cell count or proportion.

The analysis of **Figure 10** reveals that none of the immune cell abnormalities (NK cells, B cells, total T lymphocytes, Th cells, Tc/Ts cells, and CD4/CD8 ratios) show significant differences in PFS probabilities. The log-rank tests for all comparisons indicate no significant differences, and the hazard ratios (HRs) suggest no strong evidence of an impact on PFS. This indicates that the presence or absence of these specific immune cell abnormalities does not significantly influence the progression-free survival outcomes in this cohort.

## Discussion

This study investigates the impact of transvascular interventional therapy on immune cell dynamics, DCR and PFS in HCC patients. A single-center observational case-control study was conducted with 119 patients who met the inclusion criteria. Various interventional treatments, including TACE, TAE, and HAIC, were administered based on patient-specific evaluations. The study revealed significant findings in immune cell dynamics and their correlation with treatment outcomes. Total WBCs counts, lymphocyte counts, monocyte counts, and basophil counts were significantly higher in the success control group compared to the failure control group, suggesting their potential role in treatment success. Neutrophils and eosinophils showed no significant differences between the groups. Subgroup analysis revealed that abnormal BMI, diabetes, infection, and multiple lesions were strongly associated with T cell abnormalities. Age, abnormal BMI, hypertension, and abnormal AFP were linked specifically to total T cell abnormalities. The results also highlighted that NK cells, B cells, Th cells, Tc/Ts cells, and CD4/CD8 ratios did not show significant differences in PFS probabilities, suggesting that these specific immune cell abnormalities do not significantly influence PFS outcomes.

Total WBC, lymphocyte, monocyte, and basophil counts were significantly higher in the success group, indicating their potential role in treatment success, while neutrophils and eosinophils showed no significant differences. Subgroup analysis revealed that abnormal BMI, diabetes, infection, and multiple lesions were associated with T cell abnormalities, suggesting these factors may affect immune response and treatment efficacy in HCC. Additionally, age, abnormal BMI, hypertension, and abnormal AFP levels were linked to total T cell abnormalities, emphasizing the importance of understanding patient-specific factors in treatment outcomes.

The results showed no significant differences in PFS probabilities for NK cells, B cells, Th cells, Tc/Ts cells, and CD4/CD8 ratios, suggesting these immune cell abnormalities do not significantly impact PFS outcomes. This indicates that while some immune cells are critical for treatment success, others may not predict long-term survival. Understanding these nuances can help create more effective and personalized treatment strategies for HCC patients. By combining interventional therapies with immune modulation, we can enhance anti-tumor responses and overcome resistance. Further research is needed to clarify the mechanisms behind these associations and improve therapeutic approaches.

The success control group has significantly higher counts of total white blood cells, lymphocytes, monocytes, and basophils compared to the failure control group. This suggests that these cell types may be linked to treatment success. In contrast, neutrophils and eosinophils show no significant difference between the two groups, indicating that they may not be key factors influencing treatment outcomes. These findings suggest that certain types of WBCs may play important roles in treatment success, necessitating further research to elucidate their specific mechanisms and clinical significance.

The significant findings of our study hold considerable implications for the treatment of HCC using interventional therapy. The higher counts of total WBCs, lymphocytes, monocytes, and basophils observed in the success control group suggest these cells play a crucial role in treatment effectiveness. These differences in immune cell dynamics indicate their importance in determining therapeutic outcomes. This highlights the potential of using immune cell profiles as biomarkers. They could be valuable for predicting treatment outcomes. The absence of significant differences in neutrophil and eosinophil counts clarifies which immune cells contribute to treatment success. The identified associations between clinical variables (abnormal BMI, diabetes, infection, multiple lesions) and T cell abnormalities highlight the complex interaction between patient factors and immune responses. These findings suggest that personalized treatment strategies considering these variables could improve therapeutic efficacy. The correlation of T cell abnormalities with age, abnormal BMI, hypertension, and AFP levels further underscores the need for personalized approaches. As confirmed by previous studies, diabetes and hypertension have a certain impact on the prognosis of HCC patients treated with sorafenib (26).

Our findings also reveal that certain immune cell abnormalities, including those in NK cells, B cells, Th cells, Tc/Ts cells, and CD4/CD8 ratios, do not significantly influence PFS. This indicates that while some immune cells are critical for treatment success, others may not be as predictive of long-term survival outcomes. Understanding these nuances is essential for developing more targeted and effective treatment regimens. The results highlight the importance of immune cell dynamics in the prognosis of HCC patients undergoing interventional therapy. The key roles of specific immune cells, along with clinical factors affecting immune responses, offer a solid basis for improving treatment strategies. As emphasized by numerous previous researchers, the significant role of the immune cell-dominated tumor microenvironment in the treatment of HCC cannot be overlooked (27–29). Further research is necessary to elucidate the mechanisms behind these associations and to enhance the clinical application of immune profiling in HCC treatment. Integrating immune modulation with interventional therapies can enhance anti-tumor responses and help overcome resistance. This approach may ultimately lead to better clinical outcomes for HCC patients.

In patients with HCC, the elevation of white blood cells, lymphocytes, monocytes, and basophils following vascular interventional therapies carries significant clinical implications. This increase indicates that interventional therapy not only targets the tumor vasculature by cutting off its blood supply but also modulates the immune environment, potentially activating the body’s natural antitumor immune response (30, 31). The rise in white blood cells and lymphocytes suggests enhanced immune surveillance and tumor clearance, while elevated monocyte levels may promote antigen presentation and further stimulate immune responses. Additionally, the increase in basophils hints at possible immune regulation, potentially working in synergy with anti-inflammatory and anti-tumor immune mechanisms (32, 33).

These changes in immune cell dynamics may also enhance the effectiveness of immunotherapies, such as PD-1/PD-L1 inhibitors, by priming the tumor microenvironment to be more immunogenic and less suppressive. By reshaping the immune landscape, therapies can reduce immune evasion mechanisms of tumors and increase the body’s natural immune response to the malignancy (34, 35). The combination of interventional and immune-based therapies could significantly improve treatment efficacy and offer more personalized therapeutic strategies for HCC patients.

While our study offers valuable insights into the impact of immune cell dynamics on HCC treatment outcomes, several limitations should be noted. First, being a single-center study, our findings may not be generalizable to other populations, necessitating future multicenter studies for broader validation. Second, the small sample size of 119 patients may affect the statistical power, requiring larger cohort studies to confirm our results. The observational nature of the study limits the ability to establish causal relationships between immune cell dynamics and treatment outcomes, highlighting the need for controlled trials. Our focus on specific immune cell subsets may have overlooked other relevant immune cell populations and molecular pathways. Comprehensive profiling of the tumor microenvironment and systemic immune responses using advanced techniques like single-cell RNA sequencing could provide a more detailed understanding of the immune landscape in HCC (36, 37). Fourth, the follow-up period of 28 days post-intervention may not capture long-term immune responses and their effects on patient outcomes. Extended follow-up studies are required to assess the durability of the immune changes observed and their long-term prognostic value. Future research should involve multicenter studies with larger cohorts and longer follow-ups. Advanced techniques and patient-specific factors will enhance personalized treatments and improve HCC outcomes.

Our study identified significant differences in immune cell dynamics between successful and unsuccessful HCC treatments by transvascular antitumor interventional therapy. Higher counts of white blood cells, lymphocytes, monocytes, and basophils were linked to treatment success, while neutrophils and eosinophils showed no significant differences. Clinical factors such as abnormal BMI, diabetes, infection, and multiple lesions correlated with T cell abnormalities, highlighting the need for personalized treatment strategies. Summarizing, these insights could help predict treatment success and optimize therapeutic approaches, improving patient outcomes by integrating immune modulation with interventional therapies.

## Data Availability

The raw data supporting the conclusions of this article will be made available by the authors, without undue reservation.
